# “Comparative analysis of predictors of failure for high-flow nasal cannula in bronchiolitis”

**DOI:** 10.1371/journal.pone.0309523

**Published:** 2024-11-21

**Authors:** Ana Carolina Etrusco Zaroni Santos, Carolina Marques Caiado, Alessandra Geisler Daud Lopes, Gabriela Cunha de França, Camila Araujo Valerio, Danielle Bruna Leal Oliveira, Orlei Ribeiro de Araujo, Werther Brunow de Carvalho

**Affiliations:** 1 Pediatric Intensive Care Unit, Hospital Municipal Infantil Menino Jesus, São Paulo, São Paulo, Brazil; 2 Parasitology Department, Universidade de São Paulo, São Paulo, São Paulo, Brazil; 3 Microbiology Department, Universidade de São Paulo, São Paulo, São Paulo, Brazil; 4 Pediatric Intensive Care Unit, GRAAC, Universidade Federal de São Paulo, São Paulo, São Paulo, Brazil; 5 Pediatric Intensive Care Unit, Instituto da Criança, Universidade de São Paulo, São Paulo, São Paulo, Brazil; AIIMS: All India Institute of Medical Sciences, INDIA

## Abstract

**Objective:**

To assess a comparative analysis of the ROX index, Wood-Downes-Ferrés score (WDF), p-ROXI, and the SpO_2_/FiO_2_ ratio as predictors of high-flow nasal cannula (HFNC) failure in children hospitalized for bronchiolitis.

**Methods:**

Data were extracted from the clinical trial “Comparison between HFNC and NIV in children with acute respiratory failure caused by bronchiolitis” conducted at a tertiary Brazilian hospital (Emergency Department and PICU). The inclusion criteria were children under 2 years of age admitted for bronchiolitis who developed mild to moderate respiratory distress and were eligible for HFNC therapy. Performance was determined by ROC and AUC metrics to define the best sensitivity and specificity for each variable. Children were evaluated at 0 h, 2 h, 6 h, 12 h, 24 h, 48 h, 72 h and 96 h after HFNC therapy initiation.

**Results:**

A total of 126 patients were recruited for this analysis. The median age was 3 months. Ninety-one percent of the patients had an identified viral agent, with RSV being the most common (65%). Twenty-three percent (29/126) of patients experienced failed HFNC therapy and required mechanical ventilation. The best cutoff points at 12 hours were 4.5 for WDF (AUC = 0.83, 0.74–0.92), 8.8 for ROX (AUC = 0.7, 0.54–0.84), 1.45 for p-ROXI (AUC = 0.56, 0.38-0-74), and 269 for SpO_2_/FiO_2_ (AUC = 0.64, 0.48–0.74). The scores and indices were also correlated with the PICU and hospital LOS.

**Conclusions:**

The ROX index and WDF were the most accurate scores for assessing HFNC failure considering 12-hour cutoff points.

**Trial registration number:**

U1111-1262-1740; RBR-104z966s. Date of registration: 03/01/2023.

## Introduction

Acute viral bronchiolitis is the most common lower respiratory disease and the primary reason for hospitalization among infants. According to a survey carried out in the United States between 2010 and 2019, approximately 20% of hospitalized patients required intensive care, 4.9% required noninvasive mechanical ventilation, and 3.3% required invasive mechanical ventilation [1, 2].

For patients who experience acute respiratory failure, high-flow nasal cannula (HFNC) therapy has been shown to be an effective alternative for ventilatory support [1, 3, 4]. However, in the case of therapy failure, delaying tracheal intubation may worsen the prognosis. In addition, the tracheal intubation criteria differ in the literature, making ideal time determination a challenge. In this context, the application of predictive HFNC therapy failure scores, including cutoff values, could be useful for guiding clinical decision-making [1, 5–7].

The respiratory rate-oxygenation (ROX) index was developed by Roca et al. as a predictor of success in adults with pneumonia and is defined as the SpO_2_/FiO_2_/RR ratio. This index has been analyzed mainly in adult population. The new findings suggest the ROX index as a predictor of HFNC failure and outcome. However, some concerns were raised considering its accuracy and aplicability [8–15]. In pediatrics, publications confirming the ROX index and its variations applicability are scarce and have multiple limitations [16–18]. In a recent study, Yildizdas and collaborators adjusted the ROX index formula for children between one month and eighteen years of age considering the normal variation in respiratory frequency according to age group (p-ROXI). The results were correlated with therapeutic success, although the study could not define a cutoff failure point. Furthermore, most of the patients recruited had congenital heart disease, which impaired its external validation in the general pediatric population [16].

The Wood–Downes–Férres score (WDF) was initially developed in the 1970s to assess children with asthma exacerbation [19]. Subsequently, it was modified and applied for patients with bronchiolitis to assess severity and therapeutic response [20]. Among neonates hospitalized for bronchiolitis, a decrease in the score seems to reflect a therapeutic response to HFNC therapy. However, this finding has not yet been demonstrated in older children [21].

The applicability of the SpO_2_/FiO_2_ ratio has been extensively analyzed in adults and children. This tool is useful for evaluating HFNC patients’ response noninvasively. Nonetheless, there are few prospective studies comparing its accuracy with that of other scores [22–25].

Therefore, the objective of this study was to compare the performance of the ROX index, WDF score, SpO_2_/FiO_2_ ratio and p-ROXI as predictors of HFNC failure in infants hospitalized for acute viral bronchiolitis.

## Materials and methods

The following data were extracted from the clinical trial titled “Comparison between high-flow nasal cannula and noninvasive ventilation (NIV) in children with acute respiratory failure caused by bronchiolitis” carried out between February 9^th^ 2021 and May 3^rd^ 2023 in the Emergency Department and Pediatric Intensive Care Unit of Hospital Municipal Infantil Menino Jesus (HMIMJ), São Paulo, Brazil. This is a 92-bed tertiary pediatric hospital that comprises a 20-bed pediatric intensive care unit (PICU) with a monthly average admission rate of approximately 70 patients.

The primary analysis is under structuring for publication. In the present study, patients with a diagnosis of mild to moderate acute respiratory failure due to bronchiolitis were randomized into two groups according to sealed envelopes (HFNC or NIV). Those who experienced therapy failure were intubated. In an interim analysis, the failure rate was 32% (17 of the 52 randomized patients). Therefore, we estimated that a sample of at least 87 patients would be representative of this population for the current study, with a margin of error of 10% and a 95% confidence interval. This study was approved by the Institutional Review Board (IRB) of the hospital (Hospital Municipal Infantil Menino Jesus Research Ethics Committee; approval number 39509820.0.0000.5639, 11/24/2020; trial registration number: U1111-1262-1740; RBR-104z966s, 03/01/2023). Despite clinical trial submission had been occurred before patient randomization, its registration was approved later because some formatting and data adjustments were required. All procedures were performed in accordance with the ethical standards of the responsible committee and the Declaration of Helsinki. Written informed consent was obtained from the children’s parents or guardians before the data were collected and was applied by the physician responsible for starting the protocol. The STROBE guidelines were applied for text structuring considering the score comparison [26].

Local training was carried out with physicians and physiotherapists before and during the research for device usage and protocol application. Training efficacy evaluation was not performed.

The HFNC system used was an Airvo 2 (Fisher & Paykel), and the initial flow was 2 L.kg.min. FiO_2_ was titrated to maintain a peripheral oxygen saturation between 94 and 99%. All eligible patients who developed mild to moderate respiratory distress (classified by WDF score) were connected to a nonrebreathing mask until the device was properly installed.

The inclusion criteria were children under 2 years of age admitted for bronchiolitis that progressed to mild to moderate respiratory distress (WDF < 8) during hospitalization [27, 28]. The exclusion criteria were patients with severe respiratory distress, bronchopulmonary dysplasia, cyanotic congenital heart disease or hemodynamic repercussion; liver disease; neuromuscular disease; or tracheostomy. Infants with a viral panel positive for severe acute respiratory syndrome coronavirus 2 (SARS-CoV-2) and whose parents refused to participate in this study were also excluded.

The ROX index, WDF score, SpO_2_, FiO_2_ and vital signs were registered at 0 h, 2 h, 6 h, 12 h, 24 h, 48 h, 72 h and 96 h after HFNC therapy initiation. The variables assessed included age, weight, sex, comorbidities, viral panel identified by reverse transcription polymerase chain reaction (RT–qPCR), time on HFNC, mechanical ventilation duration, PICU length of stay (LOS), hospital LOS, and mortality. Data in S1 Table and S1 Appendix provide a description of the variables applied in each score.

The criteria for indicating HFNC failure and mechanical ventilation requirements were signs of severe respiratory distress (WDF 8 to 14), a respiratory rate > 60 bpm (in children up to 1 year old) or > 40 bpm (in children 1 to 2 years old), or a heart rate > 160 bpm. For patients who experienced respiratory improvement, weaning therapy was administered according to the institutional protocol.

As a primary outcome, the ROX index, WDF, SpO_2_/FiO_2_ and p-ROXI, which are predictors of HFNC failure, were evaluated. The secondary outcomes analyzed were mechanical ventilation duration, PICU LOS, hospital LOS, and mortality.

The descriptive statistics included measures of central dispersion (means and standard deviations, medians, and interquartile ranges), in addition to absolute and relative frequencies.

The Mann–Whitney U test was used to compare distributions. To evaluate correlations, the Spearman test was used, and to compare frequencies, the chi-square test was used. Receiver operating characteristic (ROC) curves and the Youden index were used to identify the best cutoff points for predicting HFNC therapy failure and determining the sensitivity and specificity of the cutoff points. The ROC curves were compared through the DeLong test. Generalized estimating equations (GEEs) were applied to analyze repeated measures of the ROX index and WDF. Statistical analysis was performed with R (Vienna, Austria) [29].

The p-ROXI was calculated according to Yildizdas (SpO_2_/FiO_2_/respiratory rate z score for age). Respiratory rate z scores for age were obtained from Sepanski et al. [16, 30].

The interrater reliability of the WDF was not evaluated. The standard method was applied for missing data, which is the analysis of complete cases, not using any technique of replacement or imputation [31]. The percentages of missing data for each period were 0 h (0), 2 h (0.2%), 6 h (0.8%), 12 h (0.9%), 24 h (0.1%), 48 h (0.7%), 72 h (0) and 96 h (0). Missing data occurred as a result of incomplete forms.

## Results

**Fig 1:**
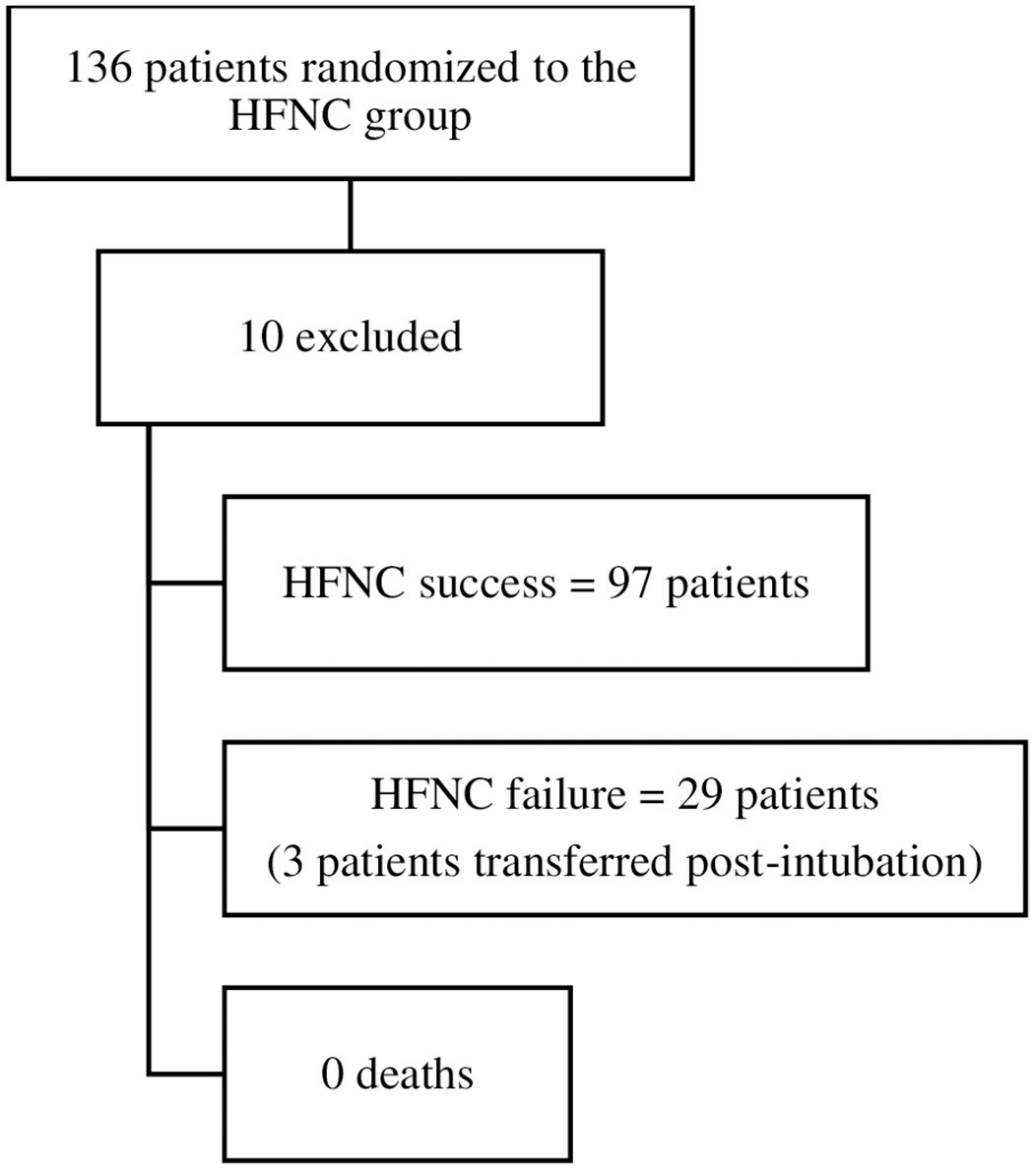
Study flowchart.

A total of 126 patients were randomized to the high-flow nasal cannula group and were included in this analysis. Fig 1 shows the study flowchart.

Table 1 presents the demographic and clinical course variables, descriptive statistics of the failure and success groups, and comparisons between the means when applicable.

**Table 1:** Demographic and clinical characteristics.

	Intubated (n = 29)		Nonintubated (n = 97)		p*
Age (months, median, IQR)	3	2–4	3	2–7	0.223
Weight for age (z score, median, SD)	-0.21	0.93	-0.11	1.12	0.471
HFNC duration (hours, median, IQR)	9	6–25	91	70–120	0.001
PICU LOS (days, median, IQR)	8	5–10	5	4–6	0.001
MV duration (days, median, IQR)	4	3–4.8	-	-	-
Hospital LOS (days, median, IQR)	11.5	8–13.8	7	6–9	0.001
Antibiotics (n, %)	19	65	30	30.1	0.002
**Comorbidities**					
Atopic dermatitis (n, %)	-	-	1	1%	-
Prematurity 35–37 weeks (n, %)	-	-	4	4.1%	-
Epilepsy	-	-	1	1%	-
Cleft palate	-	-	1	1%	-
Patent foramen ovale	-	-	3	3%	-
Subglottic stenosis	-	-	1	1%	-

*p = Mann‒Whitney U test values

SD = standard deviation

IQR = Interquartile range

A total of 126 randomized patients (HFNC group) participated in this analysis after 10 were excluded. The exclusion criteria were failure to fully comply with the protocol, a hospitalization for non-respiratory reason, prematurity less than 35 weeks, a diagnosis of heart disease with hemodynamic repercussions during hospitalization, external transfer during HFNC therapy, and a positive viral panel for SARS-CoV-2. There was no occurrence of air leak syndrome or death. Twenty-nine patients (23%) failed HFNC therapy and required invasive mechanical ventilation. Patients who responded to HFNC therapy had a longer duration of therapy (91 ± 70–120 hours versus 9 ± 6–25 hours, p < 0.001), shorter PICU LOS (5 ± 4–6 days versus 8 ± 5–10 days, p < 0.001), shorter hospital length of stay (7 ± 6–9 days versus 11.5 ± 8–13.8 days, p < 0.001) and fewer antibiotic requirements (30.1 versus 65%, p = 0.002).

The number of intubated patients according to temporal evolution was 0 h (0), 2 h (1), 6 h (6), 12 h (11), 24 h (4), 48 h (5), 72 h (1), and 96 h (1).

Table 2 presents vital signs and scores in the first 24 hours of HFNC therapy. There were no statistically significant differences in the initial vital signs, indices, or scores between the failure and success groups. During this period, the variable that showed a sustained difference in the assessments was the WDF score (at 2 h, 6 h, 12 h, or 24 h).

**Table 2:** First 24-hour vital signs and scores.

	Nonintubated		Intubated		p*
	Mean	SD	Mean	SD	
Initial HR (bpm)	153.7	15.8	158.2	18.0	0.174
Initial RR (bpm)	51.0	11.5	54.0	12.3	0.241
Initial SpO_2_ (%)	97.5	1.9	96.8	1.8	0.073
Initial WDF	5.9	0.9	6.0	0.9	0.872
Initial ROX	6.0	2.3	5.3	1.7	0.235
Initial SpO_2_/FiO_2_	291.9	76.0	273.6	61.8	0.201
Initial FiO_2_ (%)	36.0	11.2	37.1	8.4	0.261
Initial flow (l.min)	13.5	3.7	13.1	4.3	0.374
Initial p-ROXI	2.9	5.1	2.2	8.3	0.418
HR 2 h (bpm)	141.8	17.2	152.3	19.6	**0.006**
RR 2 h (bpm)	45.5	12.1	50.6	10.6	**0.009**
SpO_2_ 2 h (%)	97.8	1.6	97.4	1.9	0.359
WDF 2 h	5.0	1.1	5.8	1.2	**0.002**
ROX 2 h	6.9	2.7	5.7	1.9	**0.022**
SpO_2_/FiO_2_ 2 h	292.8	80.1	270.3	62.5	0.161
FiO_2_ 2 h (%)	36.0	10.7	37.8	8.2	0.175
Flow 2 h (l.min)	13.4	3.7	12.8	4.1	0.264
p-ROXI 2 h	2.4	8.6	0.6	7.0	**0.043**
HR 6 h (bpm)	138.8	15.9	147.8	19.8	**0.025**
RR 6 h (bpm)	43.1	9.3	46.3	16.8	0.232
SpO_2_ 6 h (%)	98.1	1.7	96.8	2.7	**0.021**
WDF 6 h	4.7	1.1	5.5	1.7	**0.031**
ROX 6 h	7.6	2.7	7.4	5.0	0.211
SpO_2_/FiO_2_ 6 h	310.6	78.9	275.5	71.4	0.113
FiO_2_ 6 h (%)	33.7	8.9	37.6	10.3	0.179
Flow 6 h (l min)	13.3	3.5	13.6	4.8	0.741
p-ROXI 6 h	3.1	12.8	6.5	11.3	0.489
HR 12 h (bpm)	138.8	18.1	146.7	16.1	0.158
RR 12 h (bpm)	42.9	9.8	51.1	12.4	**0.023**
SpO_2_ 12 h (%)	97.2	4.7	97.3	3.8	0.481
WDF 12 h	4.6	1.2	6.2	1.1	**0.001**
ROX 12 h	7.9	2.9	5.8	2.5	**0.023**
SpO_2_/FiO_2_ 12 h	316.5	93.2	269.7	84.7	0.101
FiO_2_ 12 h (%)	32.2	8.6	41.0	19.4	0.061
Flow 12 h (l min)	13.3	3.6	12.7	4.7	0.344
p-ROXI 12 h	1.7	11.4	2.3	4.0	0.482
HR 24 h (bpm)	139.1	18.9	158.6	15.2	**0.014**
RR 24 h (bpm)	42.3	8.2	47.7	9.0	0.072
SpO_2_ 24 h (%)	98.2	2.1	98.4	2.1	0.718
WDF 24 h	4.3	1.4	5.7	0.8	**0.004**
ROX 24 h	8.8	2.7	6.7	1.3	**0.032**
SpO_2_/FiO_2_ 24 h	359.1	74.9	311.6	59.4	0.092
FiO_2_ 24 h (%)	28.6	6.2	32.6	6.1	0.111
Flow 24 h (l min)	12.9	3.9	11.1	3.2	0.212
p-ROXI 24 h	3.2	10.4	0.9	4.7	0.121

*p = Mann–Whitney U test values. It was not possible to compare patients who were intubated or not after 48 hours, as only two patients were intubated in this period.

Table 3 presents the panel viral results. Respiratory syncytial virus (RSV) was the most common viral agent identified, with no difference between the groups (75.8% versus 70.1%, p = 0.54).

**Table 3:** Viral panel (n, %).

	Intubated		Nonintubated		p
RSV	22	75.8%	68	70.1	0.542
Adenovirus	2	6.9%	7	7.2%	-
Bocavirus	1	3.4%	9	9.3%	-
Seasonal coronavirus	2	6.9%	6	6.2%	-
Parainfluenza	1	3.4%	4	4.1%	-
Metapneumovirus	1	3.4%	3	3.1%	-
Rhinovirus/enterovirus	-	-	2	2.1%	-
Negative	3	10.3%	16	16.5%	-
2 or more viruses	3	10.3%	16	16.5%	-

*p = Chi-squared test value. No comparisons were made in other categories due to low occurrences.

**Fig 2:**
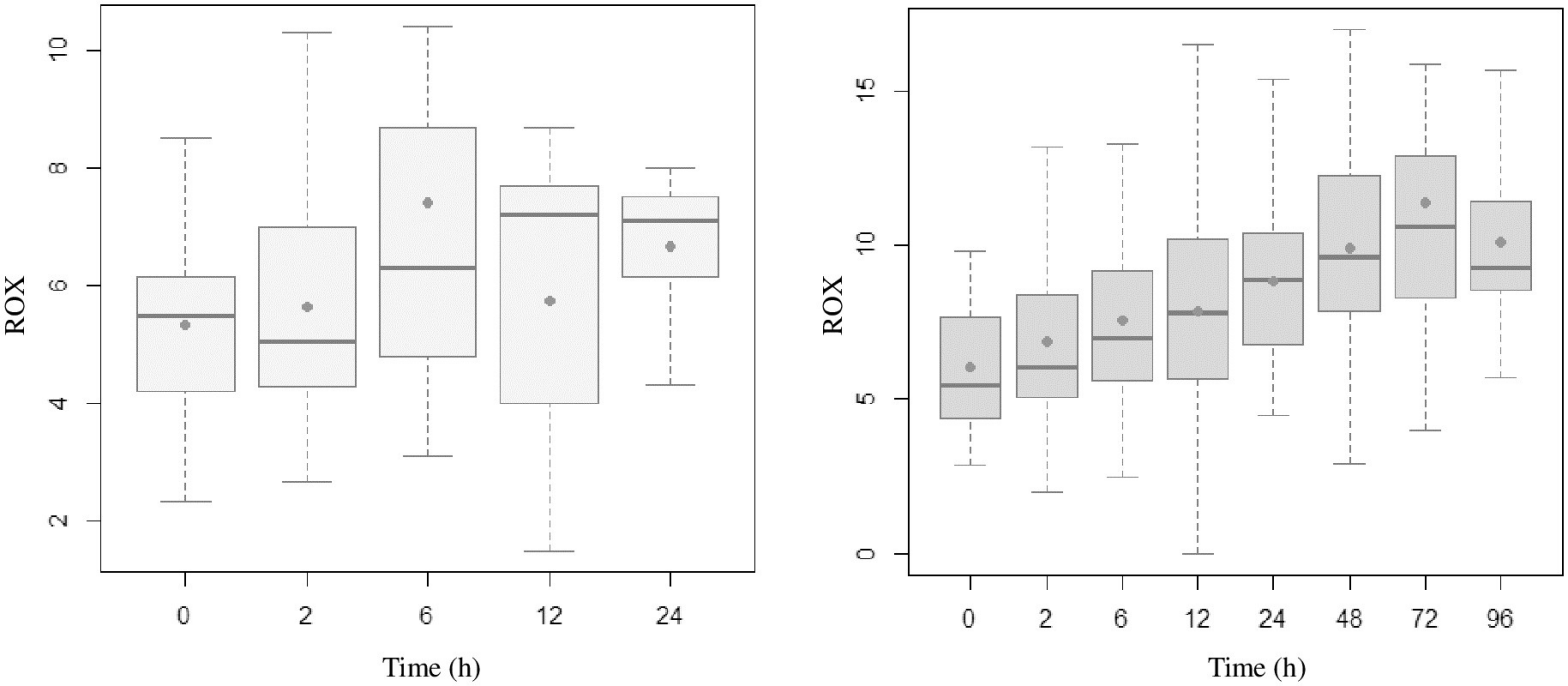
Box plots representing ROX values of patients who were intubated (up to 24 hours) and those who were not. The averages are represented by dots.

**Fig 3:**
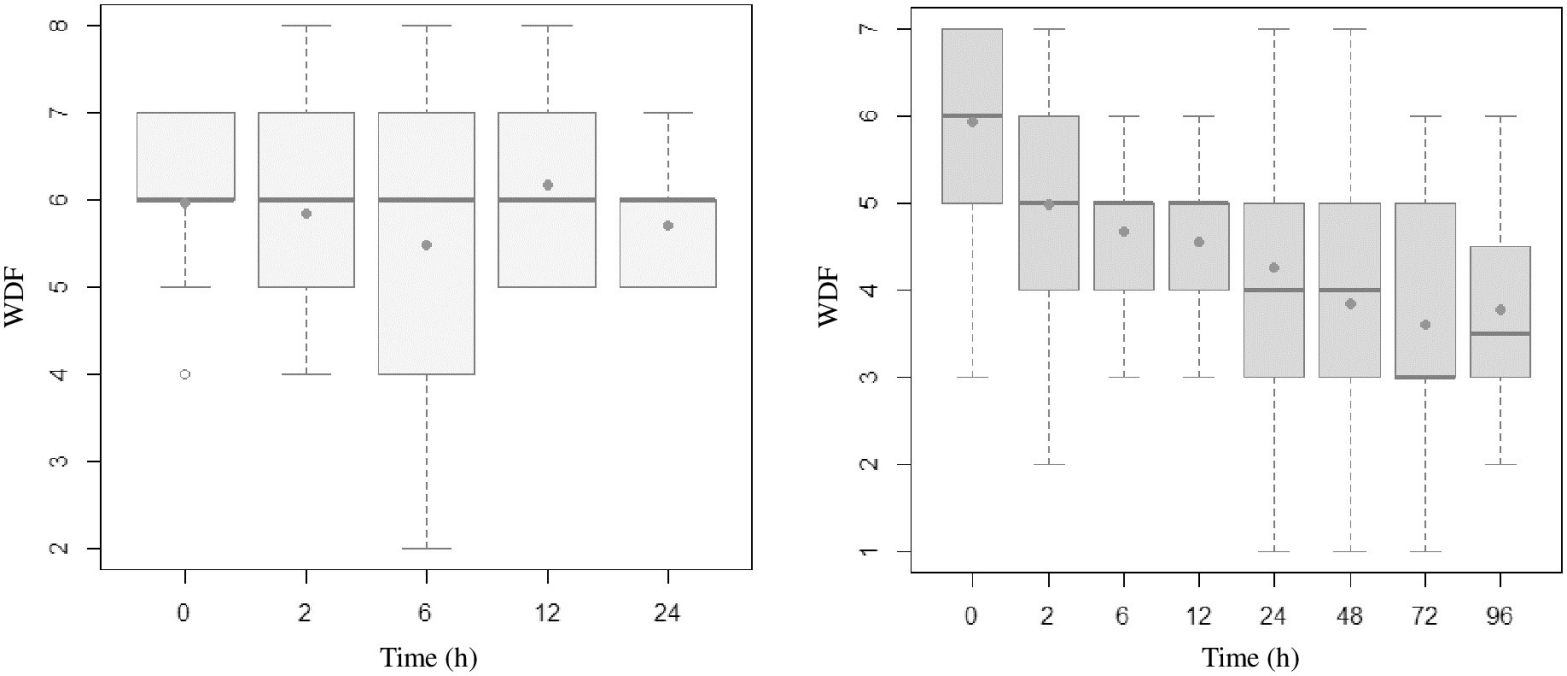
Box plots representing WDF values of patients who were intubated (up to 24 hours) and those who were not. The averages are represented by dots.

**Fig 4:**
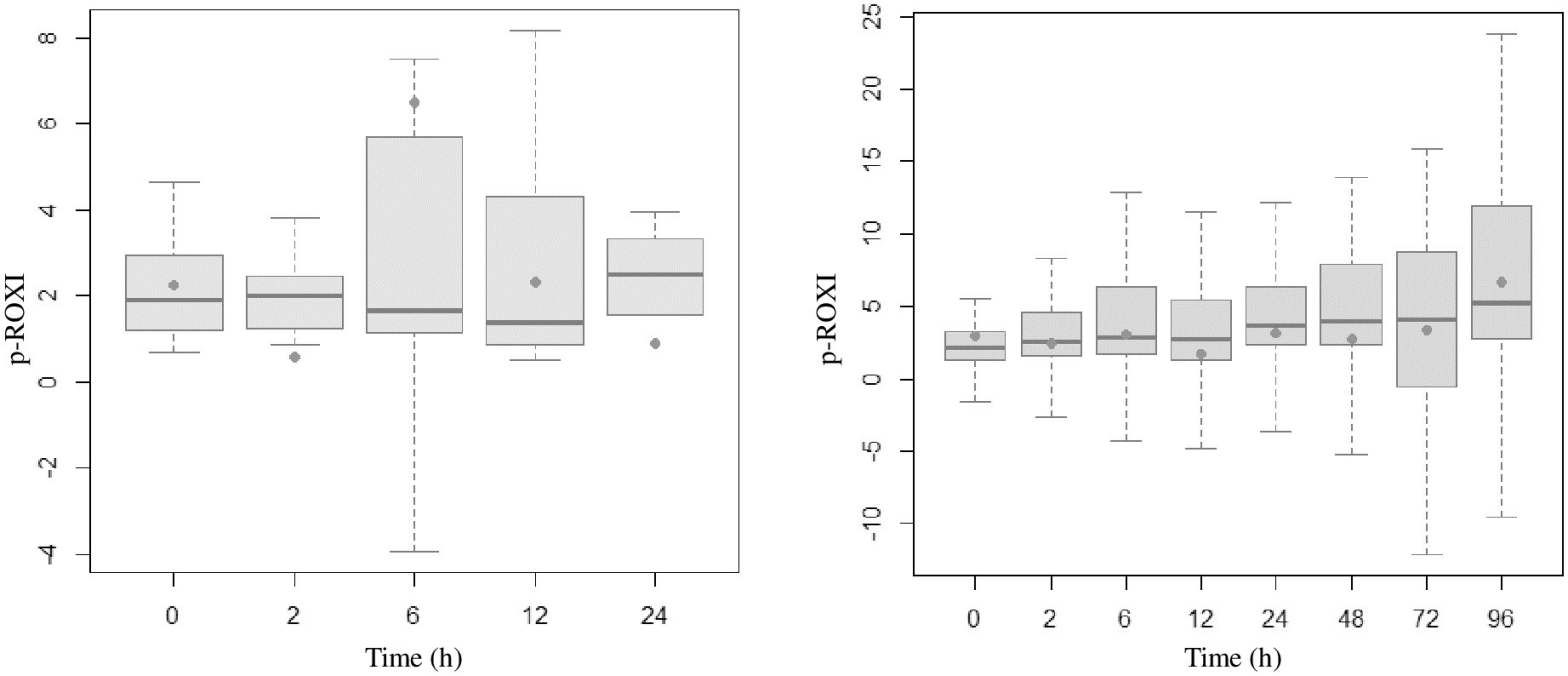
Box plots representing p-ROXI values of patients who were intubated (up to 24 hours) and those who were not. The averages are represented by dots.

**Fig 5:**
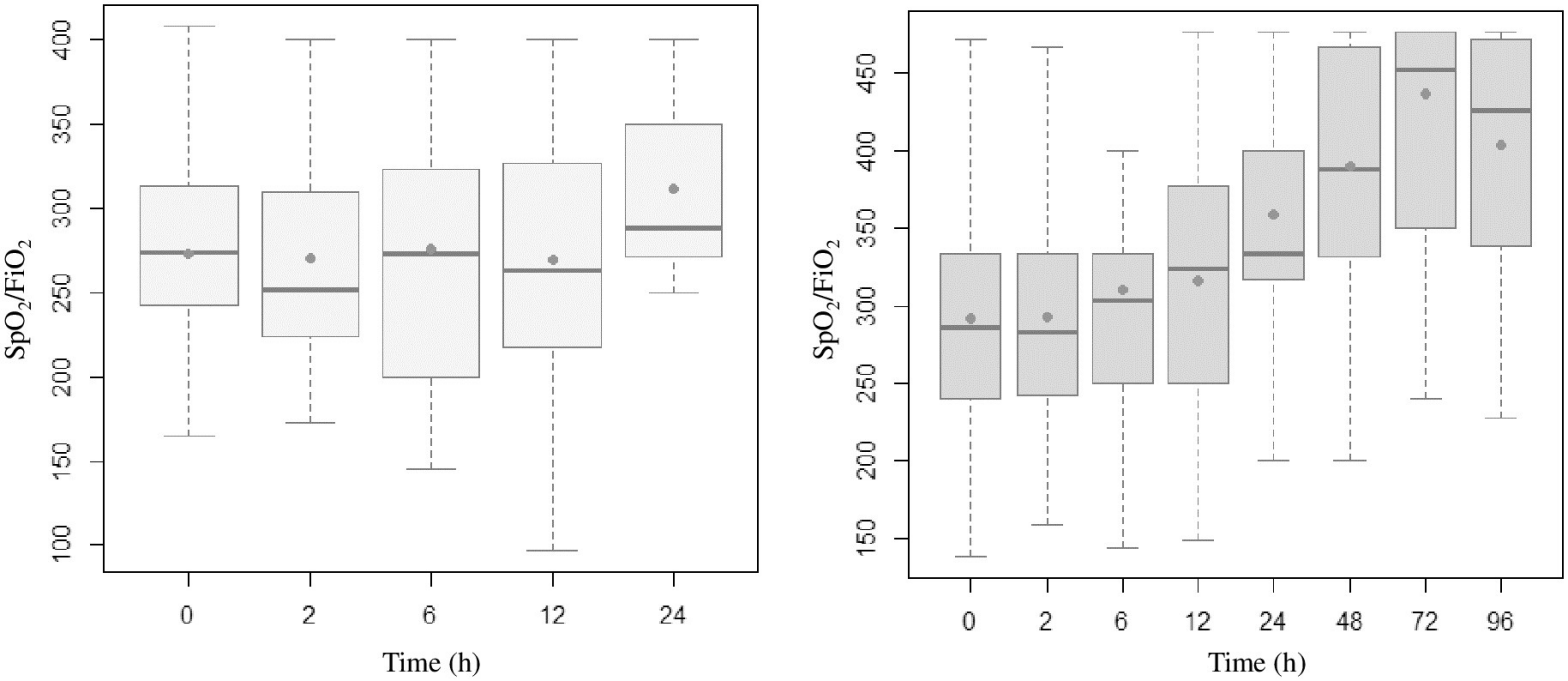
Box plots representing SpO_2_/FiO_2_ ratios of patients who were intubated (up to 24 hours) and those who were not. The averages are represented by dots.

Figs 2–5 present the temporal evolution of ROX, WDF, SpO_2_/FiO_2_ and p-ROXI by group (intubated vs. nonintubated).

Table 4 presents the area under the ROC curve (AUC) values for the ROX, WDF, SpO_2_/FiO_2,_ and p-ROXI scores; 95% confidence intervals; best score discrimination points; sensitivity; and specificity. AUC analysis was not performed for 48, 72 or 96 hours due to the reduced number of intubations in these periods (n = 7).

**Table 4:** AUC for intubation outcomes.

	AUC	95% CI	Best cutoff point	Best cutoff point sensitivity	Best cutoff point specificity
Initial WDF	0.51	0.39–0.62	5.5	76%	29%
WDF 2 h	0.69	0.57–0.8	5.5	61%	71%
WDF 6 h	0.66	0.5–0.82	5.5	58%	76%
WDF 12 h	0.83	0.74–0.92	4.5	100%	48%
WDF 24 h	0.82	0.72–0.91	4.5	100%	59%
Initial ROX	0.42	0.31–0.54	5.5	50%	49%
ROX 2 h	0.64	0.52–0.76	4.9	50%	76%
ROX 6 h	0.58	0.43–0.73	5.5	43%	76%
ROX 12 h	0.70	0.54–0.84	8.8	100%	36%
ROX 24 h	0.74	0.6–0.88	8.1	100%	55%
Initial p-ROXI	0.55	0.42–0.67	1.5	44%	69%
p-ROXI 2 h	0.63	0.52–0.74	2.9	84%	45%
p-ROXI 6 h	0.55	0.39–0.7	1.75	52%	75%
p-ROXI 12 h	0.56	0.38–0.74	1.45	54%	71%
p-ROXI 24 h	0.69	0.53–0.84	4	100%	46%
Initial SpO_2_/FiO_2_	0.58	0.46–0.69	284	69%	51%
SpO_2_/FiO_2_ 2 h	0.58	0.47–0.70	240	39%	77%
SpO_2_/FiO_2_ 6 h	0.61	0.47–0.74	207	27%	93%
SpO_2_/FiO_2_ 12 h	0.64	0.48–0.74	269	61%	68%
SpO_2_/FiO_2_ 24 h	0.69	0.49–0.89	318	71%	74%

AUC = area under the ROC curve

CI = confidence interval

**Fig 6:**
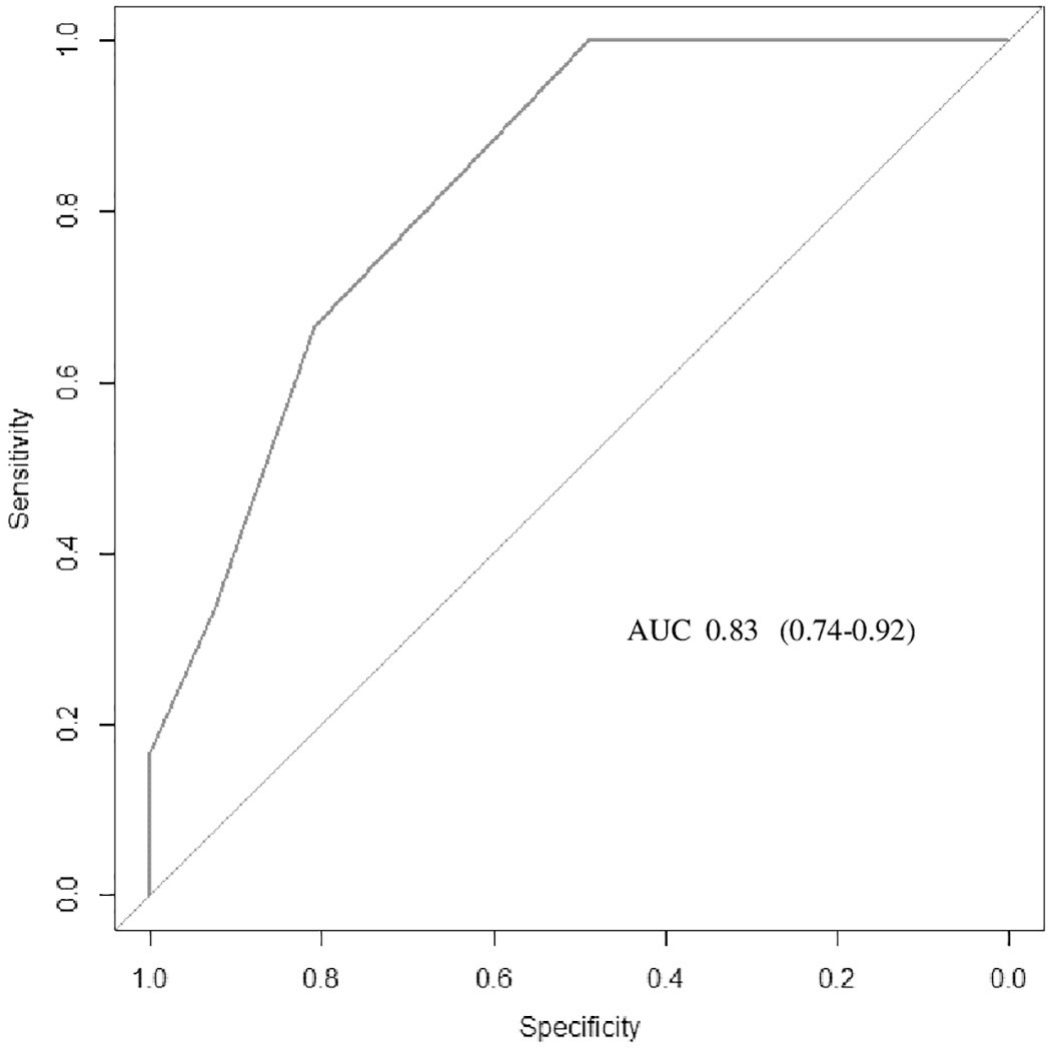
AUC of the 12-h WDF.

**Fig 7:**
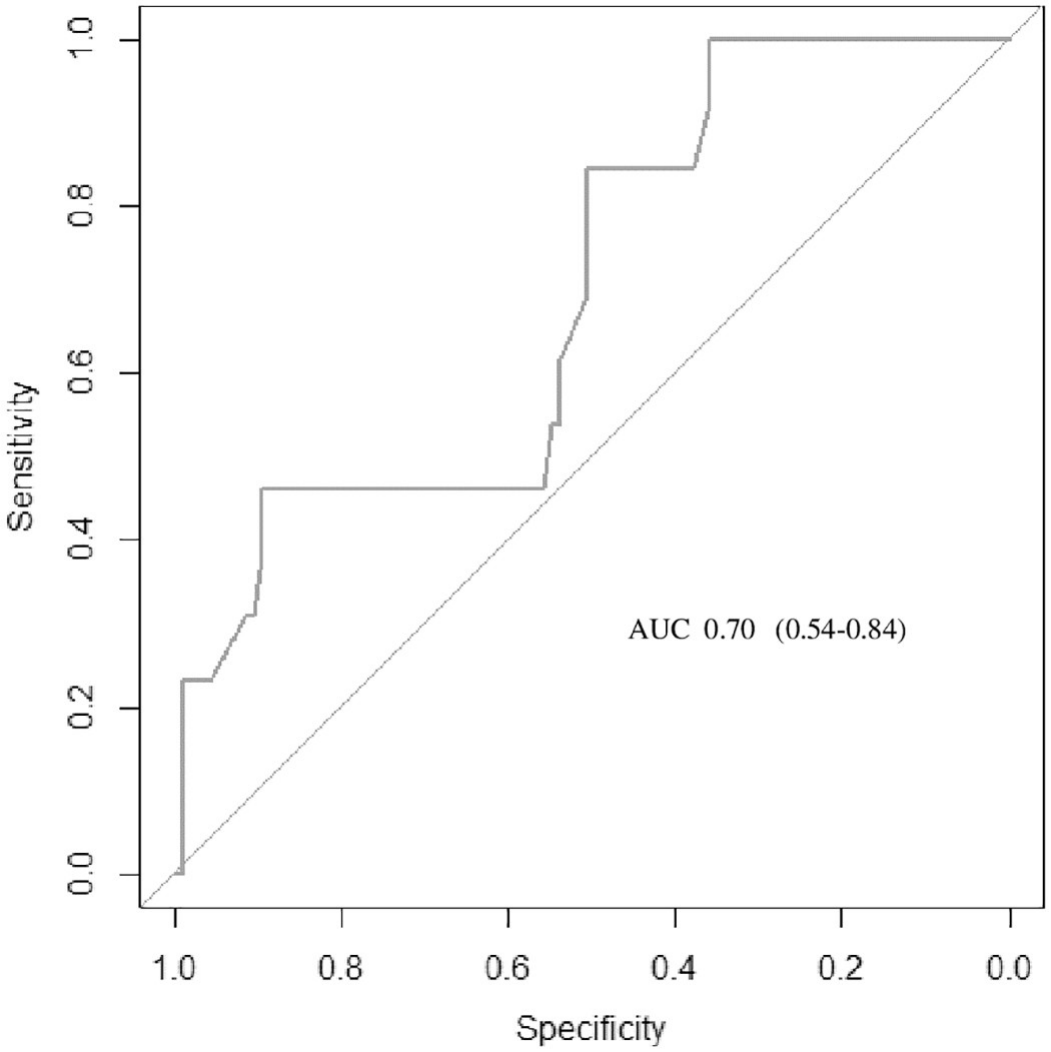
AUC of the 12-h ROX.

In general, the best AUC was obtained after 24 hours of HFNC therapy. However, considering that most intubations occurred in the first 12 hours, we analyzed this time and found that the WDF score was the best predictor of HFNC failure, with a cutoff point of 4.5 (AUC = 0.83, 0.74–0.92; 100% sensitivity and 48% specificity). At this time (12 h), the best cutoff points were 8.8 for ROX (AUC 0.7, 0.54–0.84), 1.45 for p-ROXI (AUC 0.56, 0.38-0-74), and 269 for SpO_2_/FiO_2_ (AUC 0.64, 0.48–0.74). WDF and ROX ROC analyses for 12 hours are shown in Figs 6 and 7.

The most significant ROC curves are compared in Table 5. Although the results found between the scores were different, the ROC curves were significantly equivalent between WDF at 12 h and p-ROXI at 12 h (p < 0.0001), ROX at 12 h and p-ROXI at 12 h (p = 0.03), and SpO_2_/FiO_2_ at 12 h and WDF at 12 h (p = 0.009).

**Table 5:** p values for the null hypothesis of equivalent AUC.

Scores/index	p
WDF 12 h—ROX 12 h	0.062
WDF 24 h—ROX 24 h	0.351
WDF 12 h—p-ROXI 12 h	**0.0001**
ROX 12 h—p-ROXI 12 h	**0.033**
WDF 24 h—p-ROXI 24 h	0.079
ROX 24 h—p-ROXI 24 h	0.472
SpO_2_/FiO_2_ 12 h—ROX 12 h	0.211
SpO_2_/FiO_2_ 12 h—WDF 12 h	**0.009**
SpO_2_/FiO_2_ 24 h—WDF 24 h	0.223
SpO_2_/FiO_2_ 24 h—ROX 24 h	0.578

*p = DeLong test value

AUC = area under the ROC curve

The ROX index was correlated with the PICU LOS at 24 (rho = -0.24, p = 0.02), 48 (rho = -0.40, p = 0.0001), 72 (rho = -0.33, p = 0.0005), and 96 hours (rho = -0.38, p = 0.009). For hospital LOS, there were significant correlations at 24 (rho = -0.27, p = 0.005), 48 (rho = -0.41, p < 0.001), 72 (rho = -0.28, p = 0.015), and 96 hours (rho = -0.39, p = 0.004). The ROX score was not correlated with duration of mechanical ventilation.

The WDF score was correlated with the PICU LOS at 24 (rho = 0.26, p = 0.008), 48 (rho = 0.43, p < 0.0001), 72 (rho = 0.56, p < 0.001), and 96 hours (rho = 0.49, p = 0.0006). For hospital LOS, there were significant correlations at 24 (rho = 0.27, p = 0.005), 48 (rho = 0.44, p < 0.001), 72 (rho = 0.56, p < 0.001), and 96 hours (rho = 0.41, p = 0.034). WDF was not correlated with mechanical ventilation duration.

The SpO_2_/FiO_2_ ratio was correlated with the PICU LOS at 48 (rho = -0.22, p = 0.03) and 96 hours (rho = -0.31, p = 0.03). The ratio was correlated with hospital LOS at 48 hours (rho: -0,22, p = 0.03). The SpO_2_/FiO_2_ ratio was not correlated with the duration of mechanical ventilation.

The GEE models for ROX, WDF, SpO_2_/FiO_2_, and p-ROXI are shown in Table 6.

**Table 6:** GEE model for scores/index (intubation outcomes).

	Coefficient	Standard error	OR	95% CI OR	p	Marginal mean (intubated)	Marginal mean (nonintubated)
ROX	-1.42	0.32	0.24	0.12–0.45	0.0001	6.83	8.25
WDF	1.02	0.15	2.79	2.1–3.74	0.0001	5.61	4.58
SpO_2_/FiO_2_	-0.43	0.08	0.64	0.56–0.74	0.0001	296	338
p-ROXI	0.04	0.95	1.04	0.16–6.71	0.923	2.99	2.95

OR = odds ratio

CI = confidence interval

For WDF, an odds ratio of 2.79 indicated that an increase of one unit in the score, according to repeated measurements, increased the chance of intubation by 2.79 times, which corresponded to a probability of intubation of 73% (probability = OR/1+OR; OR = odds ratio). For ROX, an OR of 0.24 indicated that an increase of one unit in the index, according to repeated measurements, reduced the chance of intubation by 76%, which corresponded to a probability of intubation of 19%. For SpO_2_/FiO_2_, an OR of 0.64 indicated that an increase of 100 units (the formula was multiplied by 100), in repeated measurements, reduced the chance of intubation by 36%, which corresponded to a probability of 39%. The p-ROXI model was not significant, with the confidence interval passing through 1.

## Discussion

The findings of this study suggest that the scores and indices analyzed could be a complementary tool in indicating ventilatory support progression in infants hospitalized for bronchiolitis who develop HFNC therapy failure. The sensitivity and specificity values varied greatly across periods, indicating that the applicability of these parameters is time dependent however possibly due to the temporal variation in the sample and the increasing prevalence of the outcome: most patients who failed HFNC therapy were already intubated within 24 hours (22/29, 75%). Considering these findings and the fundamental characteristic of a predictor of nondelaying ventilatory support progression, the best cutoff points were between 6 and 12 hours of therapy: WDF, 6 h 5.5 (AUC 0.66, CI 0.5–0.82); ROX, 6 h 5.5 (AUC 0.58, CI 1.43–0.73); p-ROXI, 6 h 1.75 (AUC 0.55, CI 0.39–0.7); and SpO_2_/FiO_2,_ 6 h 207 (AUC 0.61, CI 0.47–0.74). On the other hand, the analysis of repeated measures in the GEE showed that performing sequential evaluations of the score can be useful in assessing the probability of HFNC failure.

Most HFNC therapy failures occurred in the first 12 hours of therapy (62%, median 9 hours, SD 6–25). These results, as well as the percentage of failure (23%), are in agreement with what has already been reported [24, 25, 32].

Ruiz et al., in a retrospective analysis of 19 patients, reported a ROX value greater than 5.98 after 8 hours of therapy as a predictor of HFNC success (AUC 0.96, 95% CI 0.88–1.04) [33]. Another study among patients hospitalized for bronchiolitis demonstrated that an initial ROX less than 5.5 was a warning sign of therapy failure, indicating a more detailed evaluation requirement in this group [18]. Although the best accuracy value identified were higher than those proposed in the development of ROX index (cutoff point of 4.88), a recent multicenter ROX calibration retrospective study found a cutoff point closer to our findings (cutoff point of 7.86, 75% sensitivity and 70% specificity) [8, 12]. The application of the ROX index in children is challenging because of the wide range of normality of the respiratory rate. As the age range analyzed in our study was restricted to only neonates and infants, its applicability was possibly facilitated. However, these findings should be validated in larger populations with different respiratory diseases and reference respiratory rate values.

The WDF score was also useful for predicting HFNC failure and showed the highest accuracy at 12 hours of therapy, with a cut off of 4.5 (AUC = 0.83, 95% CI = 0.74–0.92). WDF was the only score analyzed that included clinical signs of respiratory compromise. However, respiratory rate values and especially heart rate could jeopardize the assessment of infants, considering the higher reference values for this age group. For this reason, cut off points more consistent with the population analyzed were applied in the failure criteria [20, 34]. Recently, WDF was evaluated in a cohort study of neonates hospitalized for bronchiolitis who received high-flow therapy. For responders, a reduction in vital signs and WDF scores was observed after 3 hours of treatment [21]. Despite being a score developed decades ago and widely applied, a cut off point suggesting therapy failure for patients with bronchiolitis receiving HFNC therapy has not yet been properly established.

Moreover, p-ROXI did not improve the AUC discrimination in relation to ROX or WDF. The best cutoff values were at 2 hours (2.9, AUC = 0.63, 95% CI = 0.52–0.74) and 24 hours (4, AUC = 0.69, 95% CI = 0.53–0.84). Another difficulty related to the p-ROXI is the impossibility of calculating this parameter when the z score is equal to zero (divided by zero).

The SpO_2_/FiO_2_ ratio has already been analyzed in children receiving high-flow nasal cannula therapy. Its values were shown to be good predictors when applied in emergency and pediatric intensive care units [23, 24]. According to our analysis, the SpO_2_/FiO_2_ ratio had the lowest accuracy overall. Applying a liberal SpO_2_ target (94 to 99%), we believe that this result could impact the final average SpO_2_, increasing it and consequently increasing the SpO_2_/FiO_2_ ratio. To date, it has not been possible to establish well-defined limits for SpO_2_ values in critically ill children, especially those receiving high-flow nasal cannula therapy [35, 36]. In a retrospective study carried out in a pediatric emergency department, FiO_2_ was the best predictor associated with high-flow therapy failure. According to our analysis, FiO_2_ values did not significantly differ between the success and failure groups during the first 24 hours [25].

Interestingly, different scores predicted similar results at 12 h of therapy when analyzed in pairs (WDF 12 h and p-ROXI 12 h; ROX 12 h and p-ROXI 12 h; and SpO2/FiO2 and WDF). At the bedside, this simplifies the assessment through a unique calculation.

Another finding that may be useful in indicating a more detailed and recurring assessment of patient respiratory status is the relationship between the progressive increase in the WDF score and intubation requirements. In contrast, increases in the ROX index and SpO_2_/FiO_2_ ratio are related to successful high-flow nasal cannula therapy.

The ROX index was also associated with longer PICU and hospital LOS. A similar result was found relating the WDF score and the SpO_2_/FiO_2_ ratio to the PICU LOS. It was not possible to establish a correlation between the results and duration of mechanical ventilation.

No difference was observed in the distribution of the viral panel results between the success and failure groups. Antibiotic administration was more frequent in the failure group. Although the focus of this analysis was on scores to predict HFNC failure, previous studies have demonstrated that antibiotic therapy is related to mechanical ventilation requirements in children with severe bronchiolitis. Nonetheless, these studies were carried out among patients who were receiving noninvasive ventilation. There are few publications evaluating the use of antibiotics and clinical outcomes in patients admitted for bronchiolitis who underwent HFNC therapy. In these cases, antibiotic therapy does not seem to increase the need for mechanical ventilation. However, the analyses were heterogeneous and had many limitations. New studies with high quality of evidence are needed to evaluate this association [32, 37, 38]. Nonetheless, patients on mechanical ventilation are prone to ventilator-associated pneumonia (VAP), one of the most common healthcare-associated infections in PICU. Despite the low incidence of VAP in the period analyzed (average incidence density rate of 3 VAP/1000 MV-day), the presence of an invasive device might have influenced antibiotic requirement [39].

Noninvasive serial assessment is a complementary tool whose accuracy requires constant improvement. Clinical score application for initial screening and posterior follow-up of hospitalized patients has been recommended in recent publications to standardize the evaluation [1]. However, their external validation presents many challenges, such as lower accuracy and interobserver agreement. Furthermore, the wide range of reference levels for respiratory and heart rate in children creates some barriers to establishing cutoff points and adjusting results according to a specific age group.

This study has several limitations: it was carried out in a single center and had a small sample size. The hospital is primarily a teaching institution with 24-hour resident coverage led by staff in the emergency department and PICU. The onset of HFNC therapy was determined by clinicians. Only high-flow failure criteria were established. Furthermore, for this protocol, no other ventilatory therapy support was applied (e.g. noninvasive ventilation) before tracheal intubation in the HFNC group.

Several risk factors for intubation and ICU admission (blood gas analysis, radiological changes, vasoactive usages, and bacterial coinfection) and phenotypic findings, such as recurrent wheezing, were not analyzed [1, 32]. In addition, this research was initiated during the pandemic, a period in which viral circulation and clinical presentation changed. Therefore, it is not possible to state thus far whether these findings will be just isolated points or trends that will remain [40, 41]. Another limitation is that most intubations occurred in the first 24 hours, and we could not make any assumptions about the usefulness of the score after this period.

Nevertheless, the population analyzed reflects the majority of patients treated in clinical practice—infants under 2 years old admitted to emergency and PICU environments with no comorbidities and a positive panel for respiratory syncytial virus—which makes external validation possible. Furthermore, this is an innovative study on the ROX index in pediatric patients compared with other scores. To our knowledge, no similar serial prospective analysis, including the identification of cutoff points for HFNC therapy failure, has been published.

## Conclusion

The results of this analysis suggest that the ROX index, WDF, p-ROXI and SpO_2_/FiO_2_ might be complementary bedside predictors of HFNC failure in infants hospitalized for acute viral bronchiolitis. The ROX index and WDF were shown to be the most accurate scores for assessing HFNC failure considering the 12-hour cutoff points. The indication of intubation, however, might not be restricted to the value found. Clinical evolution monitoring is a relevant component of the decision-making process. Furthermore, multicenter studies and therapy in different HFNC devices are required to support the effect of these markers in predicting HFNC therapy failure in children. Since the analysis carried out was based on the comparison between predictive power, new studies are necessary to evaluate the correlation between its application and changes in clinical outcome.

## Supporting information

S1 Table Wood-Downes-Ferres score. (DOCX)

S1 Appendix Formulas. (DOCX)

## References

[1] Milési C, Baudin F, Durand P, Emeriaud G, Essouri S, Pouyau R, et al. Clinical practice guidelines: management of severe bronchiolitis in infants under 12 months old admitted to a pediatric critical care unit. Intensive Care Med. 2023 Jan;49(1):5–25. doi: 10.1007/s00134-022-06918-4 36592200

[2] Pelletier JH, Au AK, Fuhrman D, Clark RSB, Horvat C. Trends in bronchiolitis ICU admissions and ventilation practices: 2010–2019. Pediatrics. 2021 Jun;147(6):e2020039115. doi: 10.1542/peds.2020-039115 33972381 PMC8785748

[3] Florin TA, Plint AC, Zorc JJ. Viral bronchiolitis. Lancet. 2017 Jan 14;389(10065):211–24. doi: 10.1016/S0140-6736(16)30951-5 27549684 PMC6765220

[4] Silver AH, Nazif JM. Bronchiolitis. Pediatr Rev. 2019 Nov;40(11):568–76. doi: 10.1542/pir.2018-0260 31676530

[5] Carroll CL, Napolitano N, Pons-Òdena M, Iyer NP, Korang SK, Essouri S. Second Pediatric Acute Lung Injury Consensus Conference (PALICC-2) of the Pediatric Acute Lung Injury and Sepsis Investigators (PALISI) Network. Noninvasive respiratory support for pediatric acute respiratory distress syndrome: from the Second Pediatric Acute Lung Injury Consensus Conference. Pediatr Crit Care Med. 2023 Feb 1;24(12 Suppl 2):S135–S147. doi: 10.1097/PCC.0000000000003165 36661442

[6] Bauer PR, Gajic O, Nanchal R, Kashyap R, Martin-Loeches I, Sakr Y, et al. Association between timing of intubation and outcome in critically ill patients: A secondary analysis of the ICON audit. J Crit Care. 2017 Dec;42:1–5. doi: 10.1016/j.jcrc.2017.06.010 28641231

[7] Kangelaris KN, Ware LB, Wang CY, Janz DR, Zhuo H, Matthay MA, et al. Timing of intubation and clinical outcomes in adults with acute respiratory distress syndrome. Crit Care Med. 2016 Jan;44(1):120–9. doi: 10.1097/CCM.0000000000001359 26474112 PMC4774861

[8] Roca O, Messika J, Caralt B, García-de-Acilu M, Sztrymf B, Ricard JD, et al. Predicting success of high-flow nasal cannula in pneumonia patients with hypoxemic respiratory failure: The utility of the ROX index. J Crit Care. 2016 Oct;35:200–5. doi: 10.1016/j.jcrc.2016.05.022 27481760

[9] Roca O, Caralt B, Messika J, Samper M, Sztrymf B, Hernández G, et al. An index combining respiratory rate and oxygenation to predict outcome of nasal high-flow therapy. Am J Respir Crit Care Med. 2019 Jun 1;199(11):1368–76. doi: 10.1164/rccm.201803-0589OC 30576221

[10] Gupta M, Nguyen S, Manek G, Datta D. Predicting outcomes in COVID-19 pneumonia with acute respiratory failure using the respiratory rate-oxygenation index. Chest. 2021 Oct;160(4):a1122–3. doi: 10.1016/j.chest.2021.07.1030

[11] Prakash J, Bhattacharya PK, Yadav AK, Kumar A, Tudu LC, Prasad K. ROX index as a good predictor of high flow nasal cannula failure in COVID-19 patients with acute hypoxemic respiratory failure: A systematic review and meta-analysis. J Crit Care. 2021 Dec;66:102–8. doi: 10.1016/j.jcrc.2021.08.012 34507079 PMC8424061

[12] Brewster R, Mathias S, Sarvode S, Unnikrishnan D, Raman D, Foy B, et al. A pragmatic calibration of the ROX index to predict outcome of nasal high-flow therapy in India. J Crit Care. 2024 Apr 11;82:154812. doi: 10.1016/j.jcrc.2024.154812 38608348

[13] Hill NS, Ruthazer R. Predicting outcomes of high-flow nasal cannula for Acute Respiratory Distress Syndrome. An index that ROX. Am J Respir Crit Care Med. 2019 Jun 1;199(11):1300–02. doi: 10.1164/rccm.201901-0079ED 30694696 PMC6543722

[14] Blez D, Soulier A, Bonnet F, Gayat E, Garnier M. Monitoring of high-flow nasal cannula for SARS-CoV-2 severe pneumonia: less is more, better look at respiratory rate. Intensive Care Med. 2020 Nov;46(11):2094–2095. doi: 10.1007/s00134-020-06199-9 32737522 PMC7393342

[15] Karim HMR, Esquinas AM. Success or failure of high-flow nasal oxygen therapy: the ROX index is good, but a modified ROX index may be better. Am J Respir Crit Care Med. 2019 Jul 1;200(1):116–117. doi: 10.1164/rccm.201902-0419LE 30896964 PMC6603054

[16] Yildizdas D, Yontem A, Iplik G, Horoz OO, Ekinci F. Predicting nasal high-flow therapy failure by pediatric respiratory rate-oxygenation index and pediatric respiratory rate-oxygenation index variation in children. Eur J Pediatr. 2021 Apr;180(4):1099–106. doi: 10.1007/s00431-020-03847-6 33078280

[17] Webb LV, Chahine R, Aban I, Prabhakaran P, Loberger JM. Predicting high-flow nasal cannula therapy outcomes using the ROX-HR index in the Pediatric ICU. Respir Care. 2022 Jul 19;respcare.09765. doi: 10.4187/respcare.09765 35853705

[18] Kannikeswaran N, Whittaker P, Sethuraman U. Association between respiratory rate oxygenation index and need for positive pressure ventilation in children on high flow nasal cannula for bronchiolitis. Eur J Pediatr. 2022 Nov;181(11):3977–83. doi: 10.1007/s00431-022-04607-4 36102995 PMC9525568

[19] Wood DW, Downes JJ, Lecks HI. A clinical scoring system for the diagnosis of respiratory failure. Preliminary report on childhood status asthmaticus. Am J Dis Child. 1972 Mar;123(3):227–8. doi: 10.1001/archpedi.1972.02110090097011 5026202

[20] Rivas-Juesas C, Rius Peris JM, García AL, Madramany AA, Peris MG, Álvarez LV, et al. A comparison of two clinical scores for bronchiolitis. A multicentre and prospective study conducted in hospitalised infants. Allergol Immunopathol (Madr). 2018 Jan-Fev;46(1):15–23. doi: 10.1016/j.aller.2017.01.012 28629673

[21] Barrezueta LB, Carbonell NG, Montes JL, Zafra RG, Reina PM, Herrmannova J, et al. High flow nasal cannula oxygen therapy in the treatment of acute bronchiolitis in neonates. An Pediatr (Barc). 2016 Jan. doi: 10.1016/j.anpedi.2016.03.001 27068070

[22] Emeriaud G, López-Fernández YM, Iyer NP, Bembea MM, Agulnik A, Barbaro RP, et al. Executive Summary of the Second International Guidelines for the Diagnosis and Management of Pediatric Acute Respiratory Distress Syndrome (PALICC-2). Pediatr Crit Care Med. 2023 Feb 1; 24(2)143–68. doi: 10.1097/PCC.0000000000003147 36661420 PMC9848214

[23] Saelim K, Thirapaleka B, Ruangnapa K, Prasertsan P, Anuntaseree W. Predictors of high-flow nasal cannula failure in pediatric patients with acute respiratory distress. Clin Exp Pediatr. 2022 Dec;65(12):595–601. doi: 10.3345/cep.2022.00241 36457201 PMC9742760

[24] Er A, Çağlar A, Akgül F, Ulusoy E, Çitlenbik H, Yılmaz D, et al. Early predictors of unresponsiveness to high-flow nasal cannula therapy in a pediatric emergency department. Pediatr Pulmonol. 2018 Jun;53(6):809–15. doi: 10.1002/ppul.23981 29528202

[25] Betters KA, Gillespie SE, Miller J, Kotzbauer D, Hebbar KB. High flow nasal cannula use outside of the ICU; factors associated with failure. Pediatr Pulmonol. 2017 Jun;52(6):806–12. doi: 10.1002/ppul.23626 27870384

[26] von Elm E, Altman DG, Egger M, Pocock SJ, Gøtzsche PC, Vandenbroucke JP, et al. The Strengthening the Reporting of Observational Studies in Epidemiology (STROBE) statement: guidelines for reporting observational studies. J Clin Epidemiol. 2008 Apr;61(4):344–9. doi: 10.1016/j.jclinepi.2007.11.008 18313558

[27] Sociedade Brasileira de Pediatria (SBP). Diretrizes para o manejo da infecção causada pelo vírus sincicial respiratório (VRS) 2017 [cited 2020 Aug 15]. In: SBP website [Internet]. Avaiable from: https://www.sbp.com.br/fileadmin/user_upload/Diretrizes_manejo_infeccao_causada_VSR2017.pdf.

[28] Ralston SL, Lieberthal AS, Meissner HC, Alverson BK, Baley JE, Gadomski AM, et al. Clinical practice guideline: the diagnosis, management, and prevention of bronchiolitis. Pediatrics. 2014 Nov;134(5):e1474–502. doi: 10.1542/peds.2014-2742 25349312

[29] R Foundation for Statistical Computing [cited 2020 Jul 20] [Internet]. Viena. Avaiable from: https://www.r-project.org/about.html.

[30] Sepanski RJ, Godambe SA, Zaritsky AL. Pediatric vital sign distribution derived from a multi-centered emergency department database. Front Pediatr 2018 Mar 23;6:66. doi: 10.3389/fped.2018.00066 29629363 PMC5876311

[31] Karahalios A, Baglietto L, Carlin JB, English DR, Simpson JA. A review of the reporting and handling of missing data in cohort studies with repeated assessment of exposure measures. BMC Med Res Methodol. 2012 Jul 11;12:96. doi: 10.1186/1471-2288-12-96 22784200 PMC3464662

[32] Marlow RK, Brouillette S, Williams V, Lenihan A, Nemec N, Lukowski JD, et al. Risk Factors Associated with Mechanical Ventilation in Critical Bronchiolitis. Children (Basel). 2021 Nov 11;8(11):1035. doi: 10.3390/children8111035 34828749 PMC8618830

[33] Artacho Ruiz R, Artacho Jurado B, Caballero Güeto F, Cano Yuste A, Durbán García I, García Delgado F, et al. Predictors of success of high-flow nasal cannula in the treatment of acute hypoxemic respiratory failure. Med Intensiva (Engl Ed). 2021 Mar;45(2):80–7. doi: 10.1016/j.medin.2019.07.012 31455561

[34] Fleming S, Thompson M, Stevens R, Heneghan C, Plüddemann A, Maconochie I, et al Normal ranges of heart rate and respiratory rate in children from birth to 18 years of age: a systematic review of observational studies. Lancet. 2011 Mar 19;377(9770):1011–8. doi: 10.1016/S0140-6736(10)62226-X 21411136 PMC3789232

[35] Shein SL, Karsies T. Conservative versus liberal oxygenation targets for children admitted to PICU. Lancet. 2023;S0140-6736(23)02301-2. doi: 10.1016/S0140-6736(23)02301-2 38048792

[36] Peters MJ, Gould DW, Ray S, Thomas K, Chang I, Orzol M, et al. Conservative versus liberal oxygenation targets in critically ill children (Oxy-PICU): a UK multicentre, open, parallel-group, randomised clinical trial. Lancet. 2023 Dec 1;S0140-6736(23)01968-2. doi: 10.1016/S0140-6736(23)01968-2 38048787

[37] Guitart C, Alejandre C, Bobillo-Perez S, Girona-Alarcon M, Sole-Ribalta A, Cambra FJ, et al. Risk factors and incidence of invasive bacterial infection in severe bronchiolitis: the RICOIB prospective study. BMC Pediatr. 2022 Mar 17;22(1):140. doi: 10.1186/s12887-022-03206-4 35300645 PMC8926890

[38] Leonie Lewis, Fernandes R, Kapitein B, Davies J, Holden J, Messahel S, et al. Predicting failure of high flow nasal cannula in bronchiolitis: a systematic review. European Respiratory Journal. 2019 Sep;24:Suppl. 63. doi: 10.1183/13993003.congress-2019.PA1005

[39] Antalová N, Klučka J, Říhová M, Poláčková S, Pokorná A, Štourač P. Ventilator-Associated Pneumonia prevention in pediatric patients: narrative review. Children (Basel). 2022 Oct 9;9(10):1540. doi: 10.3390/children9101540 36291475 PMC9600673

[40] Rodríguez-Fernández R, González-Martínez F, Perez-Moreno J, González-Sánchez MI, de la Mata Navazo S, Toledo Del Castillo B, et al. Clinical phenotype of respiratory syncytial virus bronchiolitis before and during the Coronavirus Disease 2019 pandemic. Am J Perinatol. 2022 Dec 21. doi: 10.1055/s-0042-1759602 36543242

[41] Bottau P, Liotti L, Laderchi E, Palpacelli A, Calamelli E, Colombo C, et al. Something is changing in viral infant bronchiolitis approach. Front Pediatr. 2022;10:865977. doi: 10.3389/fped.2022.865977 35498813 PMC9047867

