# Supplementary material for: “Comparative analysis of predictors of failure for high-flow nasal cannula in bronchiolitis”
Source: PLoS One. 2024 Nov 21;19(11):e0309523. doi: 10.1371/journal.pone.0309523 (PMC11581261; doi:10.1371/journal.pone.0309523)
Supplement: S1 Table — (DOCX) [file pone.0309523.s001.docx]

**S1 Table. Wood-Downes-Ferres score.**

|  | **0** | **1** | **2** | **3** |
| --- | --- | --- | --- | --- |
| **Wheezing** | No | End of expiration | Throughout expiration | Inspiration and expiration |
| **Retraction** | No | Subcostal + inferior intercostal | Previous + supraclavicular | Previous + superior intercostal + suprasternal |
| **Air inflow** | Good, symmetrical | Regular, symmetrical | Greatly diminished | Silent thorax (no wheezing) |
| **Cyanosis** | No | Yes |  |  |
| **Respiratory frequency** | < 30 | 31-45 | 46-60 | > 60 |
| **Heart rate** | < 120 | > 120 |  |  |

**Classification:**

Mild crisis: 1-3 points

Moderate crisis: 4-7 points

Severe crisis: 8-14 points
