# Supplementary material for: “Comparative analysis of predictors of failure for high-flow nasal cannula in bronchiolitis”
Source: PLoS One. 2024 Nov 21;19(11):e0309523. doi: 10.1371/journal.pone.0309523 (PMC11581261; doi:10.1371/journal.pone.0309523)
Supplement: S1 Appendix — (DOCX) [file pone.0309523.s002.docx]

**S1 Appendix. Formulas.**

ROX index: SpO2/FiO2/Respiratory rate

p-ROXI: SpO2/FiO2/Respiratory rate z-score
